# Digital Biosensing by Foundry-Fabricated Graphene Sensors

**DOI:** 10.1038/s41598-019-38700-w

**Published:** 2019-01-22

**Authors:** Brett R. Goldsmith, Lauren Locascio, Yingning Gao, Mitchell Lerner, Amy Walker, Jeremy Lerner, Jayla Kyaw, Angela Shue, Savannah Afsahi, Deng Pan, Jolie Nokes, Francie Barron

**Affiliations:** Cardea Bio Inc., San Diego, CA USA

## Abstract

The prevailing philosophy in biological testing has been to focus on simple tests with easy to interpret information such as ELISA or lateral flow assays. At the same time, there has been a decades long understanding in device physics and nanotechnology that electrical approaches have the potential to drastically improve the quality, speed, and cost of biological testing provided that computational resources are available to analyze the resulting complex data. This concept can be conceived of as “the internet of biology” in the same way miniaturized electronic sensors have enabled “the internet of things.” It is well established in the nanotechnology literature that techniques such as field effect biosensing are capable of rapid and flexible biological testing. Until now, access to this new technology has been limited to academic researchers focused on bioelectronic devices and their collaborators. Here we show that this capability is retained in an industrially manufactured device, opening access to this technology generally. Access to this type of production opens the door for rapid deployment of nanoelectronic sensors outside the research space. The low power and resource usage of these biosensors enables biotech engineers to gain immediate control over precise biological and environmental data.

## Introduction

The world is entering an inflection point in medical and biological testing with the simultaneous emergence of improved testing technology, advanced software tools, and increased expectations for quality healthcare worldwide. Organizations like the Qualcomm Tricorder XPRIZE and Gates Foundation have pushed for integrations of varied technologies in clinical tests to demonstrate potential application^1^. Traditional healthcare companies market point-of-care tools with limited test libraries^2^. In each case, complex, analyte-specific reagents and intricate protocols create a need for multiple platforms and deep biochemical or clinical expertise to replicate the capability of a central lab^3^.

There is a need for information-dense single assays that break the mold of expensive labs running colorimetric and PCR based assays^4^. Label-free measurement tools based on field-effect sensors should remove the need for most liquid reagents, decrease power requirements, and shrink the size of handheld testing devices^5^. These tools will be capable of performing a wide variety of chemical and biochemical assays built on top of a single sensor manufacturing chain, leading to lower overall cost for biological measurements.

To demonstrate and validate this approach, we have commercially produced and sold a digital biosensor based on graphene-enabled Field Effect Biosensing (FEB)^6^. These sensors can be described as a biologically specialized Ion Sensitive Field Effect Transistor (ISFET)^7^. We describe here the sensing mechanism, demonstrate a label-free capture assay, and summarize the critical manufacturing and quality control milestones met during recent sensor production.

The unique attributes that are required to build effective field effect biosensors are a combination of semiconductor behavior with chemical stability of the sensor surface in air and salt water^8^. Materials like silicon require oxide layers between the transistor channel and the environment, limiting the sensitivity of field effect sensors made using those conventional materials^5^. Materials, such as graphene, carbon nanotubes, and molybdenum disulfide have the unique combination of chemical stability and electric field sensitivity desirable to create sensitive electronic interfaces to biological molecules^9^. This has led to a dense literature covering chemical and biological sensors using these materials^10–22^. Several attempts were made to produce carbon nanotube biosensors for biomedical use in the early part of the 21^st^ century with frustrating results due to manufacturing difficulties^16^. Fabrication techniques using molybdenum disulfide have not matured sufficiently for devices to move beyond the proof of concept stage^17^.

Previously, we have shown that graphene biosensors can be reliably manufactured in research-oriented clean rooms^6^. Here, we show devices that are manufactured in commercial fabrication facilities under industrial quality control processes. During development of our sensor design and manufacturing process, we published several papers detailing the basic stability and performance of the sensors^6,23^. In particular we have previously shown sensitivity to infectious disease biomarkers at 18 ng/mL in buffer and 500 ng/mL in serum, with sufficient specificity to be insensitive to common interfering biomarkers^24^. Additionally, the applicability of these sensors outside the nanotechnology community has been demonstrated via publications by independent drug discovery and biotechnology groups^25–27^. With this publication, we demonstrate for the first time, the maturation of nanoelectronic digital biosensors through a more complete description of the sensor function coupled with demonstration of scaled manufacturing and quality control processes in a commercial environment.

**Figure 1:**
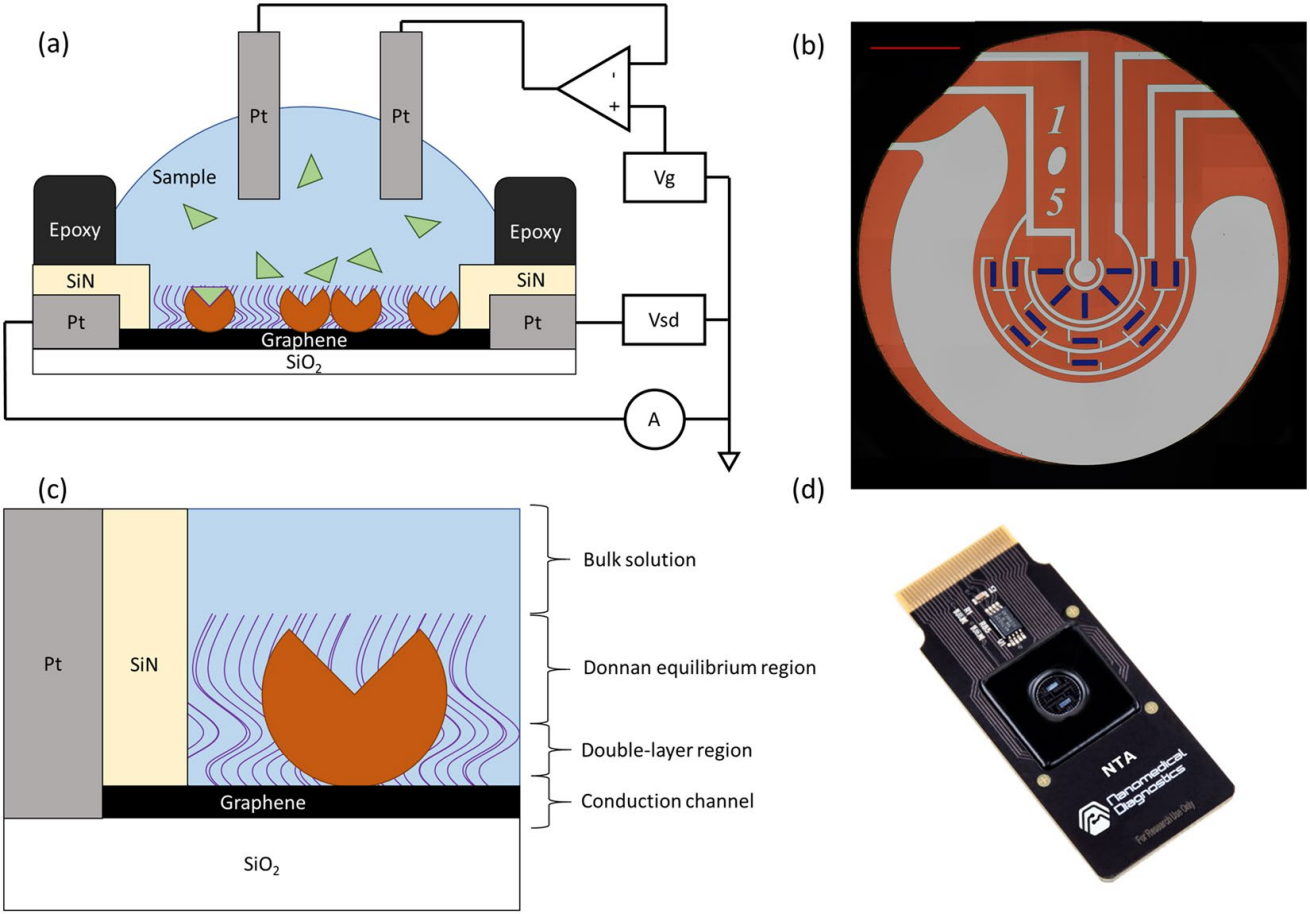
(**a**) Diagram of the sensor architecture. Circular sections on top of the graphene represent proteins embedded in a blocking layer, represented by curved lines. (**b**) A microscopy image showing an entire sensor surface. Red scalebar is 1 mm. There are fifteen graphene strips grouped into three groups of five, exposed through the silicon nitride protective layer. The center of the circuit is the gate measurement pad (pseudo-reference) and the large pad surrounding the graphene strips is the liquid gate (counter electrode). (**c**) Diagram of the sensor regions near the graphene. The double layer region is 0.3/$\sqrt{{c}_{s}}$ nm tall, where ${c}_{s}$ is the ionic strength of the bulk solution. The Donnan equilibrium region is the thickness of the combined protein and blocking layer on the surface. (**d**) Picture of the complete biosensor.

A diagram of our device architecture is shown in Fig. 1(a), and a top-down microscopy image of the active region of the biosensor is shown in Fig. 1(b). During measurement, a liquid drop is placed onto the circular region defined by the black epoxy shown here. The platinum counter and reference electrodes built into the sensor surface control and monitor a voltage in the bulk liquid. A blocking layer and embedded biomolecules such as proteins are immobilized onto 15 graphene sensors on the surface. This particular design is intended to lower the possibility that localized mechanical damage, such as from a pipette tip, would completely destroy a sensing channel. There are three sensing channels on the chip, each with five transistors spread around the surface.

The graphene sensor function relies on several overlapping effects, with a basic cartoon of the relevant layers shown in Fig. 1(c). A picture of the complete biosensor is shown in Fig. 1(d). The basic electrical function of these devices as transistors is demonstrated by an I(V_g_) graph in Supplemental Fig. 1.

## Background Theory

Closest to the graphene channel, the salts in solution will form a well-organized and well-understood layer on top of the surface countering any difference in charge between the surface and the liquid. The thickness of this layer is the Debye length and is simplified in aqueous solutions to $0.3/\sqrt{{c}_{s}}$ in nanometers, with *c*_*s*_ the ionic strength of the solution. The Donnan effect region extends beyond the Debye length, extending through the thickness of an ion-permeable membrane immobilized to the surface. The voltage in the bulk liquid is controlled by conventional electrochemical means. From an electrical perspective, the system can be understood with the bulk liquid as the gate of a transistor, and the combined Donnan region and Debye length as the dielectric between the graphene channel and the gate. From a biological perspective, the system can be understood as a voltage sensitive membrane incorporating proteins with driven voltages, like action potentials, in the bulk liquid. The model below explains how sensing is accomplished for binding interactions, even when charge transfer is not involved.

1 $$\begin{array}{c}I\approx \frac{\,W\,}{L\,}\mu \,{C}_{g}{V}_{sd}({V}_{0}-{V}_{g}+2.3\,{\varphi }_{th}\alpha {\rm{\Delta }}pH+(1-\alpha ){\rm{\Delta }}{\phi }_{D})\end{array}$$

Equation 1 shows a modification of previously developed compact models for graphene FETs when combined with ISFET models^8,28–32^. This model generally relates the current (*I*) to typical electrical properties such as the charge carrier mobility (*μ*), capacitance per unit area to the gate (*C*_*g*_), width (*W*) and length (*L*) of the graphene channel, source-drain voltage applied directly to the graphene (*V*_*sd*_) and the gate voltage (*V*_*g*_) relative to the Dirac voltage (*V*_0_). The Dirac voltage here is the gate voltage at which there is a minimum in conduction at neutral pH. The capacitance between the graene and the liquid, *C*_*g*_, is a series combination of the graphene quantum capacitance, the double layer capacitance, and a capacitance across the immobilized layer due to the Donnan effect^33,34^. It is important to note that Eq. 1 is only valid for hole conduction and does not account for non-linear gate effects near the Dirac point.

The remaining terms are corrections to the gate voltage due to the influence of pH changes and the Donnan potential. This model assumes operation of the sensor near room temperature, for an equivalent gate voltage less than the Dirac voltage, for source-drain voltages below 1 V, and for a channel length greater than 10 μm.

Electronic sensitivity to pH is typically attributed to hydrogen binding to a gate dielectric. For graphene transistors, there is no gate dielectric, but this form of pH sensitivity has been shown to apply through direct shifts in the apparent Dirac voltage^29,35^. The surface pH sensitivity factor (*α*), is a material dependent value. For clean isolated graphene, *α* is a very low 0.02, but in practice this value is increased by the presence of oxides and nitrides used in device fabrication; *α* values for practical graphene transistors are around 0.37^36–38^. This term is combined with the thermal voltage (*ϕ*_*th*_), about 26 mV, and pH shift from a neutral surface (Δ*pH*) to produce the equivalent gate voltage due to pH. Donnan potential (Δ*φ*_*D*_) is created when an ion-permeable layer separates 2 collections of ions, as shown in Eq. 2.

2 $$\begin{array}{c}\Delta {\phi }_{D}={\varphi }_{th}\,ln\frac{(\sqrt{4{c}_{s}^{2}+{c}_{x}^{2}}+{c}_{x})}{2{c}_{s}}\end{array}\,$$

In this case, the immobilized layer of proteins, peptides, surfactants, polyethylene glycol (PEG), or other soft molecules separate the graphene channel and double-layer from the bulk solution. Any charges or dipoles leading to a net charge within that immobilized ion permeable layer (*c*_*x*_) will require an extra accumulation of a counter-ion within the layer to maintain charge neutrality. This difference in the concentration of ions between the bulk solution (*c*_*s*_) and that in the immobilized protein layer creates a Donnan potential^8,39^. This additional potential enables sensing beyond the Debye screening length,^31,33,40^ and has been demonstrated repeatedly with graphene biosensors^6,15,20,24,41^.

## Results

### Chemical Measurements

This model provides a relatively simple framework for thinking about how a graphene-based digital biosensor works. Practical function of the biosensor is demonstrated by performing sensing measurements. We start by evaluating the basic response of the biosensors to common background effects such as shifts in pH and ionic strength.

**Figure 2:**
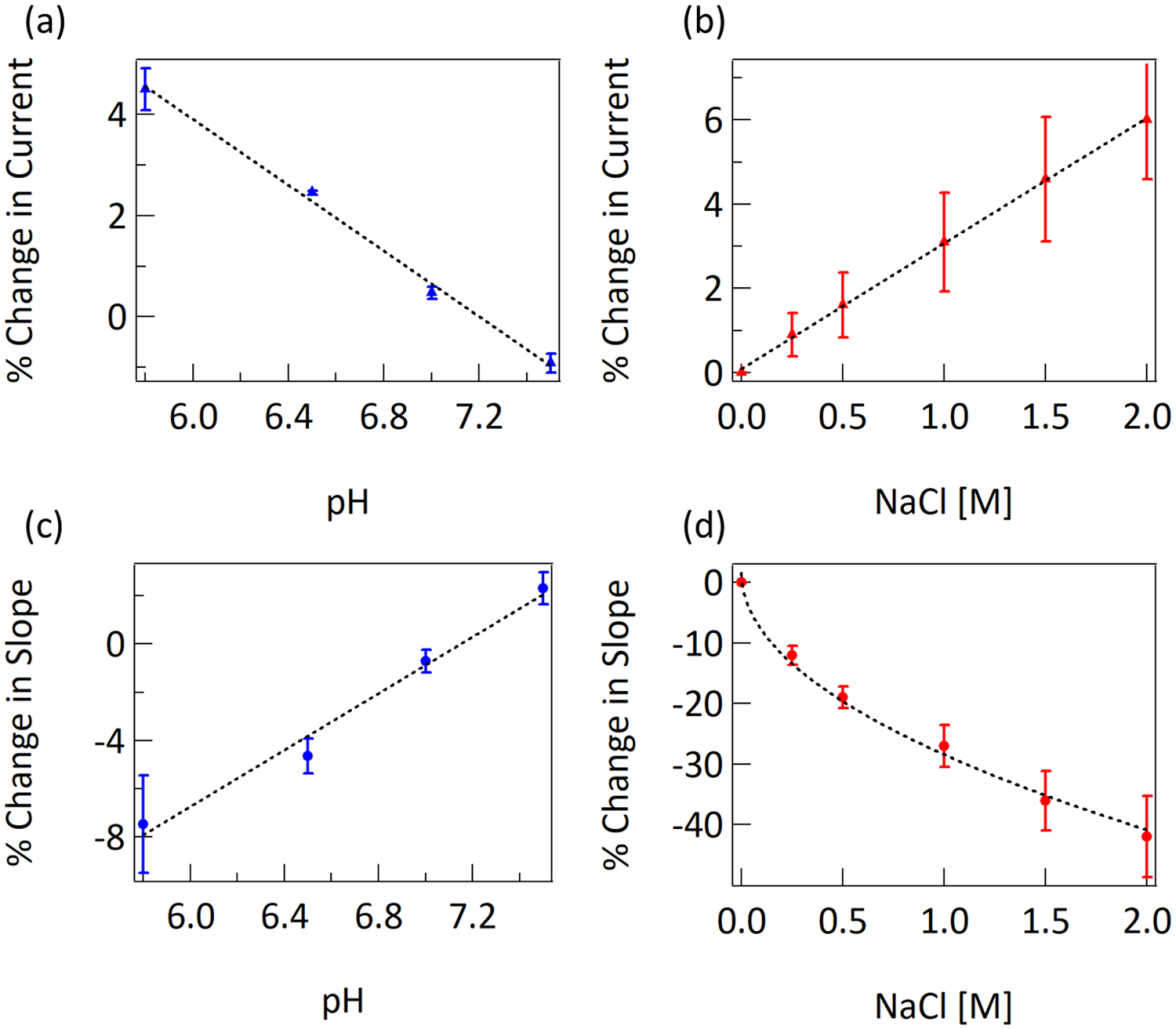
(**a**) Change in current due to change in pH. Fit is linear with a slope of −3.2% per pH unit. (**b**) Change in current due to change in ionic strength, when pH is held constant. Fit is linear with a slope of 3.0% per molar unit of NaCl. (**c**) Change in slope (dI/dVg) due to change in pH. Fit is linear with a slope of 5.9% per pH unit. (**d**) Change in slope due to change in ionic strength. Response is fit to −0.3 $\sqrt{[NaCl]}$. Each datapoint in this figure is an average of data from four sensor chips.

Our sensor data is analyzed by calculating the percent change in current from a baseline taken in assay buffer. This removes the effect of variations in resistance sensor-to-sensor. Additionally, the change in current relative to a controlled change in gate voltage Δ*I*/Δ*V*_*g*_ is used to isolate responses due to change in gate capacitance *C*_*g*_, independent of direct potential shifts from pH and Donnan potential. To generate the data in Fig. 2, the standard Agile R100 measurement settings were used, which limit applied voltages to between +/−100 mV^6,24^. The chip fabrication process purposefully creates a shift of the Dirac voltage to greater than 100 mV, so that Δ*I*/Δ*V*_*g*_ is approximately linear over all applied *V*_*g*_. This simplifies and speeds up practical biosensor measurement and analysis, while unfortunately preventing direct measurements of the Dirac voltage.

The solutions used in Fig. 2(a,c) are standard 1X phosphate buffered saline (PBS) pH 7.4 with varying amounts of 100 mM HCl added to adjust the pH. The sensors were calibrated in pH7 solutions for these measurements. The solutions used in Fig. 2(b,d) are NaCl in deionized water.

The most notable feature of the data in Fig. 2 is the dependence of the gate response slope on the square root of the ion concentration. In Eq. 1, both *α* and *C*_*g*_ are dependent on the ion concentration, but the double layer capacitance component of *C*_*g*_ is simplified to $\varepsilon \sqrt{{c}_{s}}/0.3$. The observed and modeled dependency of these sensors on pH and ionic strength indicates an ability to generate broad and complex responses, capable of creating signatures for diverse biological macromolecules and their complexes.

### Protein Measurements

**Figure 3:**
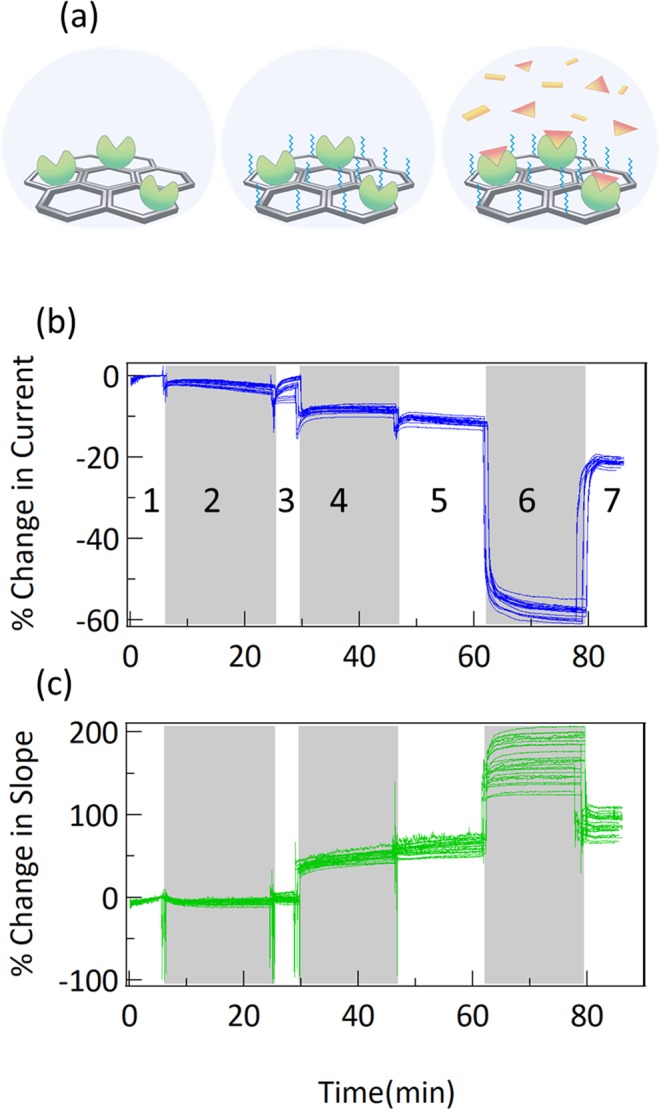
(**a**) Diagram of the steps of protein immobilization and measurement used here. First, antibodies against IL-6 are immobilized, then PEG is added as a blocker for nonspecific binding, then measurements are performed with IL-6. (**b**) Change in current and (**c**) change in slope for 23 different sensors during immobilization process. Shading is used to delineate steps: 1: calibration in MES buffer, 2: COOH activation by EDC/sNHS incubation (see methods) in MES buffer, 3: wash in MES, 4: antibody incubation in PBS, 5: PEG incubation in PBS, 6: quench in ethanolamine, 7: wash in PBS buffer.

To demonstrate measurement of a biological interaction, a monoclonal antibody against human interleukin-6 (anti-IL6) and recombinant human IL-6 (IL6) were used from a commercial ELISA kit. IL-6 is a well-known cytokine and biomarker related to inflammation, autoimmune diseases and late stage cancers. Anti-IL6 was immobilized on graphene chips with a prepared carboxyl surface and activated via standard carbodiimide chemistry (Fig. 3), as described previously^6^. Timing for each of the liquid exchange steps is described in the methods section. All liquid exchanges are done via an aspirator and manual pipette. Aspiration of liquid from the sensor surface briefly removes the liquid gate, prior to re-establishing the gate with pipetting of new liquid to the surface. This leads to the spikes in the data at the white/grey junctions.

Figure 3(b,c) show the percent change in current and slope responses for 23 different sensor chips all following the same immobilization protocol with the same anti-IL6 reagent. The relatively small variations from chip to chip highlight the stability and reproducibility of the sensors. The distribution and stability of the sensors prior to immobilization of protein is visible in Fig. 3 in step 1 (calibration) and after protein immobilization in step 7 (rinse). The manual liquid handling process used leads to slight variations in the timing, accounting for much of the variability shown in Fig. 3, and there is no background subtraction or referencing used in the presentation of this data.

The immobilization data shows consistent trends. Addition of EDC and sNHS after calibration always leads to a decrease in current, without a change in slope. This is expected as the carbodiimide chemistry should charge the charge at the surface without adding a large amount of material, and there is no adsorbed layer to create a Donnan potential. Addition of the antibody at 30 minutes leads to a further decrease in current and a large increase in slope. This change is likely due to switching from the pH 6.0 MES buffer used during carboxyl activation to the pH 7.4 PBS pH buffer used during antibody incubation. Addition of PEG-amine after anti-IL6 immobilization always leads to a small decrease in current and a small increase in slope. This is consistent with association of PEG to the surface to serve as a blocking layer around immobilized protein. After addition of PEG, a complete layer is formed on the graphene surface, enabling a Donnan effect measurement. Addition of ethanolamine (pH 8.0) quenches any remaining activated carboxyl groups, and always causes a large decrease in current and a large increase in slope. Rinsing with PBS (pH 7.4) then raises the current and decreases the slope, although never back to the initial starting position. This repeatable set of chemistry and sensor responses indicates both reproducibility of response sensor-to-sensor as well as demonstrable, permanent change to the surface chemistry of the chips due to the immobilization process. The sign (negative or positive changes) of the responses we see during immobilization are consistent with the expected responses from the pH changes measured on bare graphene chips shown in Fig. 2. However, the magnitude of responses is significantly higher.

**Figure 4:**
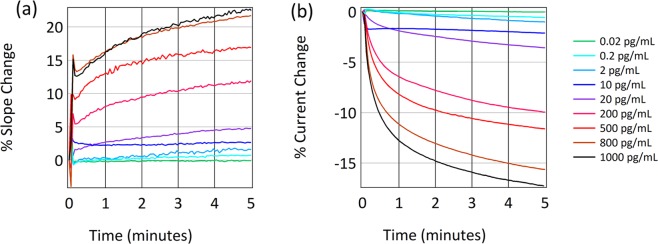
(**a**) Change in gate slope of sensors functionalized with antibodies against IL-6 to different concentrations of IL6, each measurement from a different sensor chip. (**b**) Simultaneously measured change in current.

The effectiveness of this immobilization process is demonstrated in the sensing measurements shown in Fig. 4. This is raw data, without subtraction of buffers or reference surfaces. Further development of the measurement protocols and analysis software could adopt many of the advances from the commercial biosensor field to simplify data analysis^42^. Different concentrations of IL-6 in PBS were applied to the anti-IL6 immobilized chips. The different concentrations of IL6 lead to different response magnitudes and different speeds of interaction, as expected for any kinetic binding measurement. This data compares well with the metrics provided by the ELISA kit from which the reagents were taken and previous IL-6 standard curves gathered on graphene FEB sensors^43^. It is notable that the data for 10 pg/mL has the expected magnitude after five minutes of measurement but has a shape which is different from the other responses. This may be the result of a pipetting error, variation in chip surface chemistry, or some protocol error. This is an example of the kind of information available in a real time assay that is not possible in an end-point assay. In addition, it highlights how process, user, and chip variation may be manifested in the data.

When running an ELISA with this kit, the expected sensitivity is <2 pg/mL (FEB sensitivity is <2 pg/mL), and the standard range of response is between 7.8 pg/mL and 500 pg/mL (FEB range of response is 2 pg/mL to 1000 pg/mL). As shown in Supplementary Fig. 6, performing this assay in the presence of a common interferent such as albumin does not significantly alter the results. Here, we have reproduced the ELISA quality while adding kinetics. We have removed the need for labels while providing more information-dense real-time data than an end-point assay technique like ELISA.

### Scaled Manufacturing

The data shown here establishes that the devices we have manufactured are similar in function to other biosensors in the graphene literature. A major hurdle to providing graphene biosensors to biological researchers is the typical manufacture of nanoelectronics in research-oriented clean rooms by teams of research scientists. Previously, there was no nanoelectronic manufacturing approaching ISO9001 standards or Good Manufacturing Practices (GMP), despite those requirements for commercial and clinical applications.

The facilities used to manufacture chips are located at Rogue Valley Microelectronics, Cardea, and Samtec. Rogue Valley used a 150 mm MEMS processing line, without modification to staff or equipment to process graphene wafers. The Samtec facility similarly used established silicon chip packaging equipment, staff, and traceable processes to package these graphene chips.

Graphene is grown and transferred at Cardea using automation of a previously described process^6^. The marginal cost for graphene, when using an automated system such as ours, is $0.019 per cm^2^. This comes from our average cost of copper foil of $0.012 per cm^2^ and the cost of power to run the furnaces, which in California is currently $0.007 per cm^2^ of grown graphene. This is less than the price of silicon wafer substrates for this work, which is $0.40 per cm^2^ for 150 mm wafers. The cost of processing wafers is 20-fold greater than the cost of the graphene raw material, making the processing the dominant cost factor in producing a commercial graphene device rather than the graphene raw material cost.

Our process for manufacturing digital biosensors starts with deposition and patterning of a metallization layer that creates the routing for the source-drain voltage on 15 graphene transistors per die, as well as the platinum reference and counter electrodes. Graphene is grown and transferred onto the wafer. After deposition, graphene is etched using a hard mask and an oxygen plasma. A silicon nitride encapsulation layer is deposited via plasma enhanced chemical vapor deposition (PECVD). Encapsulation here refers to deposition of a dielectric barrier layer across nearly the entire chip that is intended to prevent mechanical and chemical damage during the chip packaging and printed circuit board (PCB) assembly processes. Windows are etched into the silicon nitride down to the graphene hard mask. Wafers are diced and packaged via a chip-on-board process, where an epoxy is used to protect wirebonds and define the liquid wetted area. After packaging, the chips are annealed and undergo strict quality assurance (QA) processes to ensure reproducible quality.

**Figure 5:**
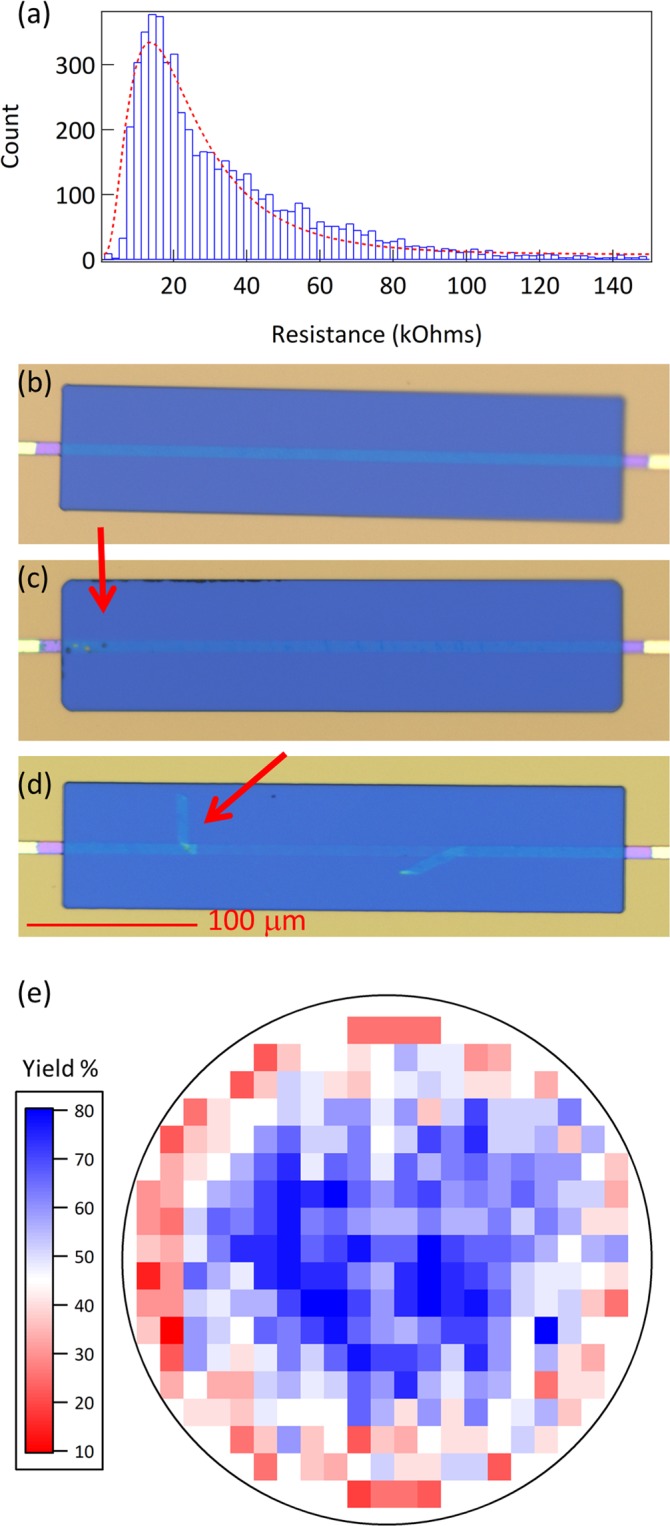
(**a**) Histogram of test resistances from 5543 chips. Fit is a log-normal distribution with a peak at 13.6 kOhm and a standard deviation of 9.2 kOhm. (**b**) Microscopy image of defect-free graphene sensor. (**c**) Microscopy image of a graphene sensor with minor polymer contamination highlighted by the red arrow. (**d**) Microscopy image of a graphene sensor with major graphene tearing highlighted by the red arrow. (**e**) Wafer yield map combining data from 27 wafers, showing cumulative % yield for each die.

Figure 5 presents quality assurance data from three production lots comprising twenty seven 150 mm wafers and 7,992 chips produced over the course of one year. Quality assurance processes focus on reproducibility and cost rather than searching for occasional outstanding performance. Specifically, this means that we use automated optical microscopy commonly found associated with commercial silicon fabrication to search for contamination and graphene tearing over entire wafers, rather than using nanotechnology specialized research hardware such as atomic force microscopy or Raman spectroscopy.

Differential interference contrast microscopy is used along with AI-enabled defect identification to perform optical evaluations, such as those shown in Fig. 5(b–d). Table 1 shows the distribution of defects found via optical microscopy across all 27 wafers. Minor defects generally do not prevent a chip from passing to the next stage of manufacture, while major defects always prevent a chip from continuing. A chip may have no defects, a single defect, or multiple defects.

**Table 1:** Defect frequency.

Issue	Number	Percent of Errors
Minor Polymer Contamination	4,066	44.2%
Minor Tears	2,857	31.0%
Major Tears	1,742	18.9%
Major Polymer Contamination	295	3.2%
Major Lithography Defect	147	1.6%
Minor Lithography Defect	77	0.8%
Other	22	0.2%

By far, the largest source of defects is polymer contamination resulting from the graphene transfer process. However, the largest source of defects that effect the operation of the chip are major tears, which may come from a variety of sources including handling, the graphene transfer process, or dust. Lithography defects are relatively rare, and primarily along the wafer edge. Other types of defects include process errors not associated with lithography, graphene double layers, or unusual forms of contamination.

In addition to focusing on use of QA tools that are current standards in commercial fabrication facilities, we also focus on optimizing the stage at which QA measurements are performed. Figure 5(a) represents the measured electrical properties of 5,543 chips out of the 7,992 which were produced. To increase efficiency, chips which fail optical QA are not measured electrically. The electrical QA data can be fit with a log normal distribution, with a peak of 13.6 kΩ ± 9.2 kΩ. The length of the measured graphene strips is 330 μm, with a width of 10 μm. Five strips are measured in parallel, for a typical resistance/sq of 1.2 kΩ/sq. Transistors that have resistances outside a range from 1 kΩ to 100 kΩ fail electrical QA. This bounds the power draw per sensor channel at between 1 nW to 100 nW when using our standard 10 mV *V*_*sd*_. This low power draw anticipates the need for thousands of chemically differentiated sensor channels or other graphene transistors in a portable form factor.

At the end of the QA process, a map of yielded dies on the wafer is produced. Figure 5(e) combines maps from 27 wafers, showing a clear bias for failure at the edge of the wafers. This likely indicates damage to the graphene transistors or lithography due to handling or normal edge effects of processing. For example, major lithography defects occur predominantly at the wafer edge. Together the data in Fig. 5(e) shows a percent yield by die position, with an average of 52%, a value that compares well with silicon device yields in the early 1980s^44^.

## Discussion

We present the first complete characterization of this fusion of electronics and biochemistry. This marks a break from the past few decades of great feats of artisanal nanoelectronics and demonstrates the potential for graphene biosensors to serve as the interface between biochemistry and digital analysis^45^.

A core challenge has been making the transition from an expensive, variable sensor to a cost-efficient, consistent batch-to-batch manufactured sensor. A key to that transition is following a rigorous specification and QA process appropriate for a biologist end user rather than a nanotechnologist. While the manufacturing and QA processes described here were used to create graphene-based biosensors, these processes are broadly enabling for many electronics applications using graphene and other nanomaterials.

The demonstrated biological sensing capability, low power requirements, and compact size of graphene-based biosensors will enable development of the next generation of biochemical applications. With the most difficult piece of the puzzle – cost-effective large-scale manufacturing – solved, low-power, portable digital biosensors can significantly impact healthcare industries with innovative new products that enable cutting-edge life science research, drug discovery applications, and diagnostic and health monitoring platforms.

## Methods

The Agile R100 system from Nanomed (a Cardea owned brand) was used for all measurements. The standard electrical settings were used. The gate voltage was swept between ±100 mV in a triangle wave at a slow speed of 0.3 Hz, while *V*_*sd*_ was held at 10 mV. An example of the raw data measured this way is shown in Supplemental Fig. 2. These voltage ranges were selected to minimize the electric fields on the proteins. Agile Plus software was used to run the hardware.

Except where noted, 1X phosphate buffered saline solution pH 7.4 (PBS) (ThermoFisher # 10010031) was used for calibration and measurement. Hydrochloric acid (HCl) was used to create PBS solutions of varying pH from 5.8 to 7.4, measured with a calibrated glass electrode pH meter. Fifty millimolar 2-(N-morpholino)ethanesulfonic acid buffer pH 6.0 (MES) (Alfa Aesar # J62574-AK, diluted in DI water) was used for crosslinking chemistry. EDC (1-ethyl-3-(3-dimethylaminopropyl)carbodiimide hydrochloride) (Amresco # N195) was used with sNHS (N-hydroxysulfosuccinimide) (G Biosciences # BC97) to activate carboxyl groups for amine attachment^46,47^. For ionic strength measurements, deionized water was used for calibration, and concentrations of NaCl from 0.25 M to 2 M in deionized water were used for the measurement. Agile FLEX biosensor chips (Nanomed) were used for pH and ionic strength measurements.

Anti-IL6 and IL6 were purchased as part of an ELISA kit (ThermoFisher # 88-7066-88).

Agile COOH biosensor chips (Nanomed) were used for IL6 measurements. COOH chips were prepared via incubation of clean graphene chips with 3 mM pyrene-carboxylic acid (TCI # P1687) in methanol for two hours.

The detailed process for anti-IL6 immobilization is: After calibration in 50 mM MES pH 6.0 for 5 minutes, 2.08 mM EDC and 5.53 mM sNHS in 50 mM MES pH 6.0 was incubated for 20 minutes. The chips were rinsed 2 times with 50 mM MES pH 6.0, followed by 14.6 nM anti-IL-6 in 1X PBS pH 7.4 for 15 minutes. Then, 3 mM PEG-amine (Broadpharm Item #BP-22355) in PBS was incubated for 15 minutes to block the surface. A blocking layer such as PEG is necessary to control non-specific surface chemistry. The chips were then incubated with 1 M ethanolamine pH 8.0 (Alfa Aesar Cat# L14322) for 15 minutes to quench any remaining carboxyl groups. A quench step is used in standard carbodiimide chemistry to remove activated carboxyl sites that may link to amines present in the test sample^6,15,24,41^. Finally, the biosensor chips were rinsed 5 times in PBS, and the last rinse was incubated for 5 minutes to stabilize the signal prior to measurement. This process was performed with a hand pipette following software prompts. Additional testing with DMEM/FBS was performed and is shown in Supplemental Fig. 5.

Prime Si, 150 mm, P-type wafers were purchased from Rogue Valley Microdevices, as was all mask fabrication and lithography. A wet thermal oxide of 3,000 Å was first grown. Metallization layers of 100 Å Cr and 500 Å Pt were patterned via liftoff technique.

Graphene was grown via chemical vapor deposition at Cardea on copper and transferred to the wafer via bubble transfer. Microscopy was used to evaluate graphene quality at every transistor location prior to further processing.

At Rogue Valley Microdevices, a 1,000 Å Au layer was deposited on the graphene and etched to form a hard mask. An oxygen plasma etch was used to remove excess graphene. A 5,000 Å SiN layer was deposited via PECVD, patterned and etched via RIE.

Custom PCBs for packaging were designed by Varasco Engineering, laid out by Pacific Design, and purchased from Consisys. Samtec diced wafers after microfabrication and performed a standard chip-on-board packaging process using gold wirebonds and a custom dam-and-fill encapsulation pattern to create the liquid well on the sensor.

The chips were cleaned, vacuum annealed at 200 °C, and inspected at Cardea.

Optical microscopy was performed using an nSPEC microscope (Nanotronics). AI driven automated defect identification software was also provided by Nanotronics.

Electrical QA was performed using an Agile R100 (Nanomed) in QA mode.

The datasets generated during and/or analyzed during the current study are available from the corresponding author on reasonable request.

## Supplementary information

Supplementary Information
