# Supplementary material for: Digital Biosensing by Foundry-Fabricated Graphene Sensors
Source: Sci Rep. 2019 Jan 22;9:434. doi: 10.1038/s41598-019-38700-w (PMC6342992; doi:10.1038/s41598-019-38700-w)
Supplement: Supplementary file 1 — Supplementary Information [file 41598_2019_38700_MOESM1_ESM.pdf]

Digital Biosensing By Foundry-Fabricated Graphene Sensors

Brett R Goldsmith*, Lauren Locascio, Yingning Gao, Mitchell Lerner, Amy Walker, Jeremy Lerner, Jayla Kyaw, Angela Shue, Savannah Afsahi, Deng Pan, Jolie Nokes, Francie Barron

Graphene Characteristic Gate Response

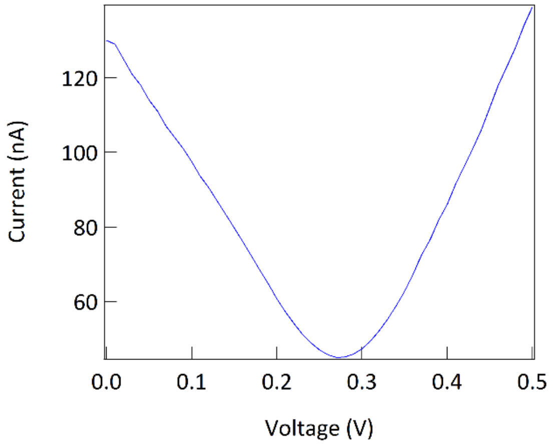

Supplemental Figure 1: An I(Vg) curve taken in 1X PBS with 10 mV source-drain showing location of the Dirac point for a representative graphene transistor.

Measurement Description

The gate voltage was swept between ± 100 mV in a triangle wave at a slow speed of 0.3 Hz, while V_{sd} was held at 50 mV. The raw data is shown in the next three figures.

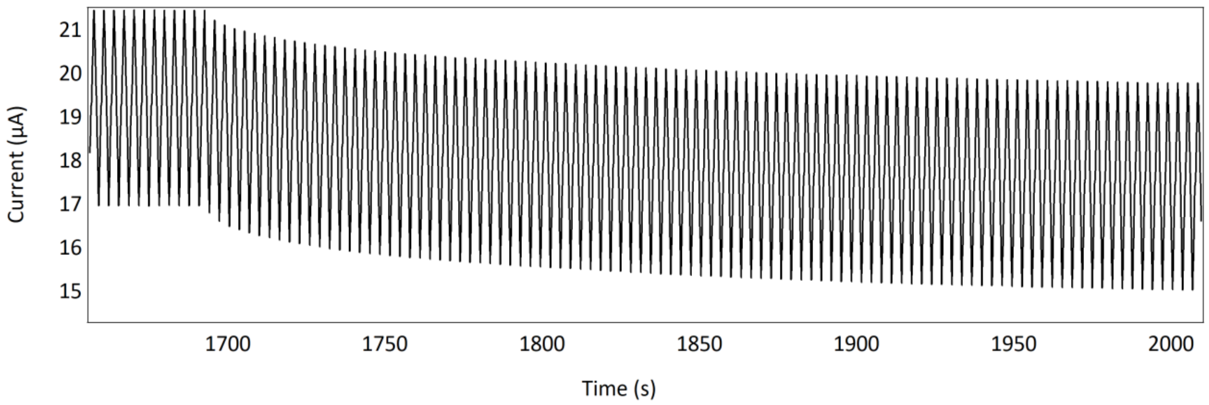

Supplemental Figure 2: Raw current data from the 1000 pg/mL curve in Figure 4 in the main text.

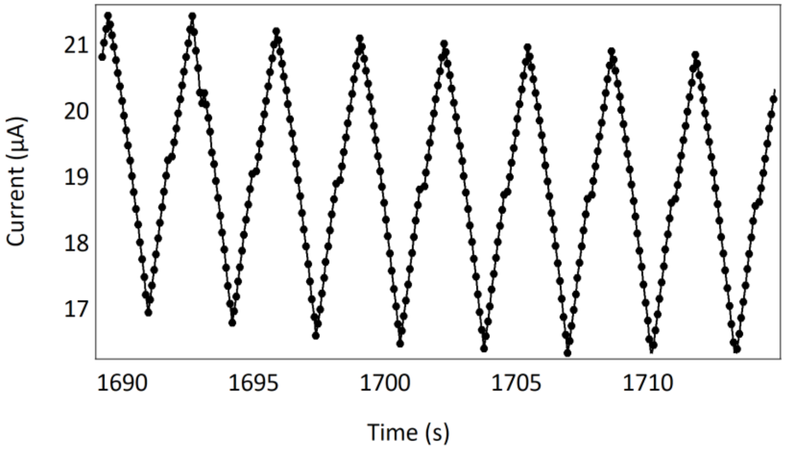

Supplemental Figure 3: Zooming in on the previous figure and resolving individual datapoints to demonstrate the speed of acquisition and the density of datapoint in a single gate curve.

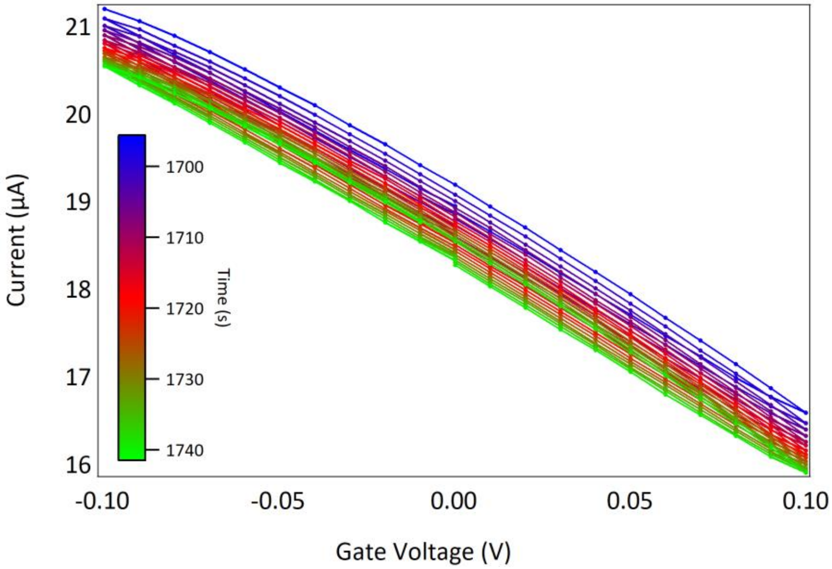

Supplemental Figure 4: The data in the previous figure can be re-plot as current versus gate, with the color indicating the time of measurement.

IL-6 Measurements in FBS

To demonstrate the effect of interfering agents on the IL-6 measurements presented in Figure 4, some simple tests were performed repeating sensing at a relatively low IL-6 concentration in Dulbecco's Modified Eagle's cell culture media containing fetal bovine serum (FBS). Cell culture requires FBS to provide the necessary components to allow for healthy cell growth. While fetal bovine albumin is the most prevalent protein in FBS, it also contains growth factors, lipids, carbohydrates, amino acids, and a variety of uncharacterized proteins. It provides an appropriate surrogate to human serum during early drug and diagnostic assay development.

The DMEM/FBS assay buffer consists of 1-part DMEM with 10% FBS diluted into 9-part 1X PBS assay buffer. Measurement was performed at 20 pg/mL of IL-6.

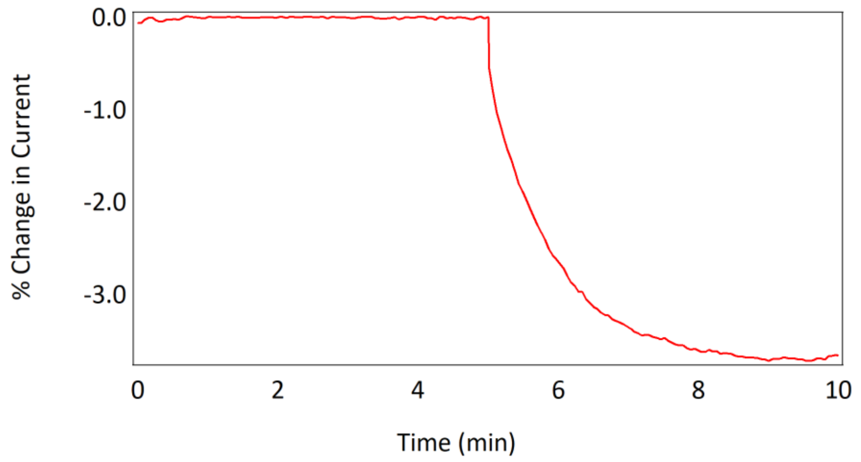

Supplemental Figure 5: Sensing curve showing response to IL-6 in DMEM/FBS. Chip was prepared as described in the main text. Calibration in DMEM/FBS was performed for 5 minutes, then was exposed to 20 pg/mL of IL-6 in DMEM/FBS.

The red curve above depicts the sensor response to 20 pg/mL IL-6 antigen in DMEM/FBS. It is important to note that the first 5 minutes of sensing data are the calibration, during which the sensor comes to equilibrium with the DMEM/FBS solution. There are many components of this solution, not the least of which is albumin, which commonly lead to large background responses for biosensors. The magnitude of the IL-6 sensing response is slightly smaller than the equivalent PBS experiment (figure 4 in the main text), although still very clear. The sensor response also reaches saturation more quickly in the DMEM/FBS buffer than in the PBS buffer.
